# Crystal structure of 3β-acet­oxy­androsta-5,16-dien-17-yl tri­fluoro­methane­sulfon­ate

**DOI:** 10.1107/S2056989015009123

**Published:** 2015-05-16

**Authors:** Shengjun Zhou, Huaqi Huang, Ting Zhang, Dangfeng Wang, Rongbin Huang

**Affiliations:** a Hangzhou Jiuyuan Gene Engineering Co. Ltd, Hangzhou 310018, Zhejiang, People’s Republic of China; b Department of Chemistry, Xiamen University, Xiamen 361005, People’s Republic of China

**Keywords:** crystal structure, chiral space group, 3β-acet­oxy­androsta-5,16-dien-17-yl tri­fluoro­methane­sulfonate, C—H⋯O inter­actions

## Abstract

The title compound, C_22_H_29_F_3_O_5_S [systematic name: (3*S*,8*R*,9*S*,10*R*,13*S*,14*S*)-10,13-dimethyl-17-(tri­fluoro­methylsulfon­yloxy)-2,3,4,7,8,9,10,11,12,13,14,15-dodeca­hydro-1*H*-cyclo­penta­[*a*]phenanthren-3-yl acetate], contains a fused four-ring steroidal system. Rings *A* and *C* adopt a chair conformation, while rings *B* and *D* adopt half-chair and envelope (with the fused CH atom as the flap) conformations, respectively. In the crystal, weak inter­molecular C—H⋯O inter­actions link the mol­ecules into layers parallel to the *ab* plane.

## Related literature   

For inhibition of the androgen signal axis in prostate cancer cells, see: Attard *et al.* (2009 ▸). For the use of the title compound as a synthetic precursor of an inhibitor of human cytochrome P450_17α_, see: Potter *et al.* (1995 ▸).
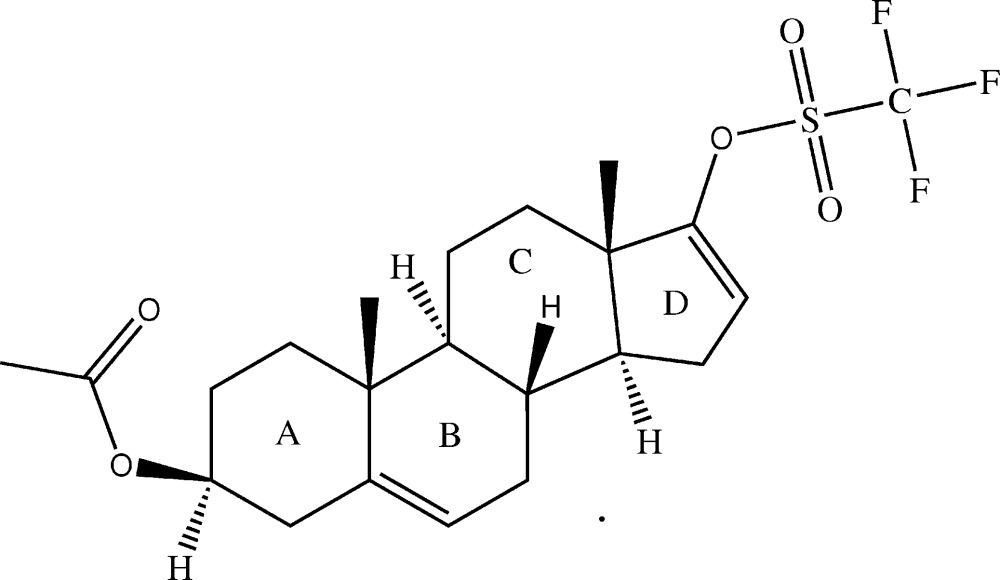


## Experimental   

### Crystal data  

C_22_H_29_F_3_O_5_S
*M* *_r_* = 462.51
Orthorhombic, 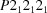

*a* = 8.0734 (10) Å
*b* = 9.9640 (12) Å
*c* = 27.6900 (15) Å
*V* = 2227.5 (4) Å^3^
*Z* = 4
Mo *K*α radiation
μ = 0.20 mm^−1^
*T* = 173 K
0.10 × 0.10 × 0.08 mm

### Data collection  

Bruker SMART APEX 2000 diffractometer
Absorption correction: multi-scan (*SADABS*; Sheldrick, 1996 ▸) *T*_min_ = 0.980, *T*_max_ = 0.984
22017 measured reflections
5098 independent reflections
3185 reflections with *I* > 2σ(*I*)
*R*_int_ = 0.058

### Refinement  

*R*[*F* ^2^ > 2σ(*F* ^2^)] = 0.060
*wR*(*F* ^2^) = 0.189
*S* = 1.11
5098 reflections
280 parameters
H-atom parameters constrained
Δρ_max_ = 0.27 e Å^−3^
Δρ_min_ = −0.66 e Å^−3^
Absolute structure: Flack *x* determined using 934 quotients [(*I* ^+^)−(*I* ^−^)]/[(*I* ^+^)+(*I* ^−^)] (Parsons *et al.*, 2013 ▸)
Absolute structure parameter: 0.02 (3)

### 

Data collection: *APEX2* (Bruker, 2004 ▸); cell refinement: *SAINT* (Bruker, 2004 ▸); data reduction: *SAINT*; program(s) used to solve structure: *SHELXS97* (Sheldrick, 2008 ▸); program(s) used to refine structure: *SHELXL2014* (Sheldrick, 2015 ▸); molecular graphics: *PLATON* (Spek, 2009 ▸) and *Mercury* (Macrae *et al.*, 2008 ▸); software used to prepare material for publication: *SHELXL2014* and *publCIF* (Westrip, 2010 ▸).

## Supplementary Material

Crystal structure: contains datablock(s) I. DOI: 10.1107/S2056989015009123/cv5486sup1.cif

Structure factors: contains datablock(s) I. DOI: 10.1107/S2056989015009123/cv5486Isup2.hkl

Supporting information file. DOI: 10.1107/S2056989015009123/cv5486Isup3.cml

**Fig. 1.**
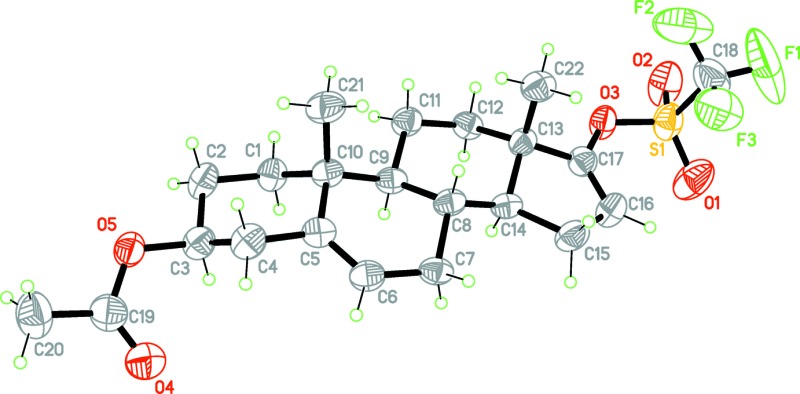
The molecular structure of (I) showing the atomic labeling and 50% probability displacement ellipsoids.

. DOI: 10.1107/S2056989015009123/cv5486fig1.tif
The mol­ecular structure of (I) showing the atomic labeling and 50% probability displacement ellipsoids.

CCDC reference: 1400503

Additional supporting information:  crystallographic information; 3D view; checkCIF report

## Figures and Tables

**Table 1:** Hydrogen-bond geometry (, )

*D*H*A*	*D*H	H*A*	*D* *A*	*D*H*A*
C1H1*A*O4^i^	0.97	2.56	3.485(6)	160
C21H21*B*O2^ii^	0.97	2.65	3.377(7)	133

Symmetry codes: (i) 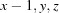; (ii) 
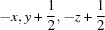
.

## supplementary crystallographic information

### S1. Structural commentary

The title compound, 3β-acet­oxy­androsta-5,16-dien-17-yl tri­fluoro­methane­sulfonate (I) (Fig. 1), is an inter­mediate of the synthesis of abiraterone acetate which is a pro-drug for 17-(pyridin-3- yl)androsta-5,16-dien-3P-ol, or abiraterone, a potent inhibitor of human cytochrome P450_17_*_α_* (steroidal 17α-hy­droxy­lase-C_17,20_-lyase) (Attard *et al.* 2009). 3β-Acet­oxy­androsta-5,16-dien-17-yl tri­fluoro­methane- sulfonate was first synthesized and charaterized by Potter *et al.* (1995), but structural data were not obtained. In this work, we obtained a single-crystal of (I) and present here its crystal structure.

The title molecule contains a fused four-ring steroidal system. The two saturated six-membered rings A and C adopt chair conformations,while ring B with one double bond adopts a half-chair conformation, and ring D with one double bond adopts an envelope conformation. The absolute structure of (I), which is crystallized in a chiral space group P2_1_2_1_2_1_, was reliably determined based on the value of Flack parameter [0.02 (3)]. In the crystal, weak inter­molecular C—H···O inter­actions link the molecules into layers parallel to *ab* plane.

### S2. Synthesis and crystallization

3β-Acet­oxy­androsta-5,16-dien-17-yl tri­fluoro­methane­sulfonate was synthesized from de­hydro-epiandrosterone acetate *via* tri­fluoro­methane­sulfonic anhydride with an overall yield of 58% according to a literature method (Potter, 1995). Colourless crystals were obtained by evaporation from a hexane solution.

### S3. Refinement

Crystal data, data collection and structure refinement details are summarized in Table 1. Crystal data, data collection and structure refinement details are summarized in Table 1. All H-atoms bound to carbon were refined using a riding model with d(C—H) = 0.93 Å, for aromatic, 0.98 Å for C—H and 0.97 Å for CH_2_ with *U*_iso_ = 1.2*U*_eq_ (C). d(C—H) = 0.96 Å with *U*_iso_ = 1.5*U*_eq_ (C) for CH_3_ H atoms. The absolute structure could be determined reliably.

### Crystal data

**Table:**

C_22_H_29_F_3_O_5_S	*F*(000) = 976
*M**_r_* = 462.51	*D*_x_ = 1.379 Mg m^−^^3^
Orthorhombic, *P*2_1_2_1_2_1_	Mo *K*α radiation, λ = 0.71073 Å
*a* = 8.0734 (10) Å	µ = 0.20 mm^−^^1^
*b* = 9.9640 (12) Å	*T* = 173 K
*c* = 27.6900 (15) Å	Block, colourless
*V* = 2227.5 (4) Å^3^	0.10 × 0.10 × 0.08 mm
*Z* = 4	

### Data collection

**Table:**

Bruker SMART APEX 2000 diffractometer	5098 independent reflections
Radiation source: Enhance (Mo) X-ray Source	3185 reflections with *I* > 2σ(*I*)
Graphite monochromator	*R*_int_ = 0.058
φ and ω scans	θ_max_ = 27.5°, θ_min_ = 3.3°
Absorption correction: multi-scan (*SADABS*; Sheldrick, 1996)	*h* = −10→10
*T*_min_ = 0.980, *T*_max_ = 0.984	*k* = −12→12
22017 measured reflections	*l* = −34→35

### Refinement

**Table:**

Refinement on *F*^2^	Hydrogen site location: inferred from neighbouring sites
Least-squares matrix: full	H-atom parameters constrained
*R*[*F*^2^ > 2σ(*F*^2^)] = 0.060	*w* = 1/[σ^2^(*F*_o_^2^) + (0.0997*P*)^2^] where *P* = (*F*_o_^2^ + 2*F*_c_^2^)/3
*wR*(*F*^2^) = 0.189	(Δ/σ)_max_ < 0.001
*S* = 1.11	Δρ_max_ = 0.27 e Å^−^^3^
5098 reflections	Δρ_min_ = −0.66 e Å^−^^3^
280 parameters	Absolute structure: Flack *x* determined using 934 quotients [(*I*^+^)-(*I*^-^)]/[(*I*^+^)+(*I*^-^)] (Parsons *et al.*, 2013)
0 restraints	Absolute structure parameter: 0.02 (3)

### Special details

**Table:**

Geometry. All e.s.d.'s (except the e.s.d. in the dihedral angle between two l.s. planes) are estimated using the full covariance matrix. The cell e.s.d.'s are taken into account individually in the estimation of e.s.d.'s in distances, angles and torsion angles; correlations between e.s.d.'s in cell parameters are only used when they are defined by crystal symmetry. An approximate (isotropic) treatment of cell e.s.d.'s is used for estimating e.s.d.'s involving l.s. planes.

### Fractional atomic coordinates and isotropic or equivalent isotropic displacement parameters (Å^2^)

**Table:**

	*x*	*y*	*z*	*U*_iso_*/*U*_eq_	
S1	−0.10597 (18)	0.04595 (17)	0.06476 (4)	0.0579 (4)	
C1	0.4181 (6)	0.0552 (5)	0.35593 (14)	0.0427 (11)	
H1A	0.3017	0.0423	0.3632	0.051*	
H1B	0.4598	−0.0284	0.3427	0.051*	
C2	0.5109 (7)	0.0846 (6)	0.40342 (16)	0.0494 (13)	
H2A	0.4642	0.1634	0.4189	0.059*	
H2B	0.4999	0.0092	0.4253	0.059*	
C3	0.6921 (6)	0.1085 (5)	0.39178 (16)	0.0449 (12)	
H3	0.7385	0.0276	0.3769	0.054*	
C4	0.7123 (7)	0.2252 (5)	0.35765 (16)	0.0468 (12)	
H4A	0.8288	0.2381	0.3505	0.056*	
H4B	0.6710	0.3064	0.3728	0.056*	
C5	0.6179 (6)	0.1994 (5)	0.31113 (16)	0.0391 (10)	
C6	0.6943 (6)	0.2032 (5)	0.26896 (16)	0.0425 (11)	
H6	0.8077	0.2195	0.2694	0.051*	
C7	0.6156 (6)	0.1838 (5)	0.22092 (16)	0.0408 (10)	
H7A	0.6479	0.0972	0.2080	0.049*	
H7B	0.6546	0.2526	0.1989	0.049*	
C8	0.4257 (5)	0.1907 (5)	0.22442 (15)	0.0372 (10)	
H8	0.3908	0.2846	0.2276	0.045*	
C9	0.3643 (5)	0.1100 (4)	0.26848 (16)	0.0366 (10)	
H9	0.4104	0.0195	0.2649	0.044*	
C10	0.4335 (6)	0.1654 (4)	0.31691 (16)	0.0374 (10)	
C11	0.1732 (6)	0.0929 (5)	0.26877 (16)	0.0420 (11)	
H11A	0.1441	0.0284	0.2936	0.050*	
H11B	0.1235	0.1781	0.2776	0.050*	
C12	0.0973 (6)	0.0464 (5)	0.22079 (15)	0.0396 (10)	
H12A	0.1297	−0.0456	0.2143	0.048*	
H12B	−0.0226	0.0498	0.2228	0.048*	
C13	0.1568 (5)	0.1370 (5)	0.17978 (15)	0.0366 (10)	
C14	0.3473 (5)	0.1296 (5)	0.17969 (16)	0.0376 (10)	
H14	0.3725	0.0336	0.1819	0.045*	
C15	0.3994 (7)	0.1698 (6)	0.12816 (16)	0.0500 (12)	
H15A	0.4076	0.2665	0.1247	0.060*	
H15B	0.5038	0.1288	0.1190	0.060*	
C16	0.2568 (7)	0.1135 (6)	0.09945 (17)	0.0519 (13)	
H16	0.2587	0.0952	0.0665	0.062*	
C17	0.1287 (6)	0.0943 (5)	0.12839 (16)	0.0413 (11)	
C18	−0.1421 (9)	0.2240 (8)	0.0518 (3)	0.081 (2)	
C19	0.9433 (6)	0.1150 (6)	0.4372 (2)	0.0546 (13)	
C20	1.0190 (8)	0.1479 (7)	0.4847 (2)	0.0683 (17)	
H20A	0.9341	0.1767	0.5067	0.102*	
H20B	1.0730	0.0697	0.4975	0.102*	
H20C	1.0987	0.2185	0.4807	0.102*	
C21	0.3391 (7)	0.2934 (5)	0.33312 (19)	0.0531 (13)	
H21A	0.2236	0.2729	0.3368	0.080*	
H21B	0.3830	0.3241	0.3634	0.080*	
H21C	0.3523	0.3623	0.3092	0.080*	
C22	0.0868 (7)	0.2809 (5)	0.18514 (18)	0.0481 (12)	
H22A	0.1028	0.3114	0.2177	0.072*	
H22B	0.1435	0.3400	0.1633	0.072*	
H22C	−0.0294	0.2806	0.1777	0.072*	
O1	0.0134 (6)	0.0001 (5)	0.03104 (14)	0.0813 (15)	
O2	−0.2654 (5)	−0.0135 (5)	0.06899 (13)	0.0779 (14)	
O3	−0.0360 (4)	0.0520 (4)	0.11675 (10)	0.0473 (8)	
O4	1.0173 (5)	0.0759 (5)	0.40211 (14)	0.0753 (13)	
O5	0.7779 (4)	0.1346 (4)	0.43747 (11)	0.0484 (8)	
F1	−0.2250 (9)	0.2339 (7)	0.0126 (2)	0.181 (3)	
F2	−0.2274 (6)	0.2797 (5)	0.0871 (2)	0.128 (2)	
F3	−0.0052 (5)	0.2913 (4)	0.04760 (17)	0.0985 (15)	

### Atomic displacement parameters (Å^2^)

**Table:**

	*U*^11^	*U*^22^	*U*^33^	*U*^12^	*U*^13^	*U*^23^
S1	0.0568 (8)	0.0737 (10)	0.0432 (6)	−0.0149 (8)	−0.0045 (6)	0.0026 (7)
C1	0.046 (3)	0.043 (3)	0.040 (2)	−0.008 (2)	0.002 (2)	0.003 (2)
C2	0.056 (3)	0.058 (3)	0.034 (2)	−0.011 (3)	0.006 (2)	−0.002 (2)
C3	0.045 (3)	0.049 (3)	0.041 (2)	−0.007 (2)	0.001 (2)	−0.005 (2)
C4	0.052 (3)	0.041 (3)	0.047 (3)	−0.006 (2)	0.002 (2)	−0.004 (2)
C5	0.037 (2)	0.033 (2)	0.047 (2)	−0.003 (2)	0.004 (2)	−0.0006 (19)
C6	0.035 (2)	0.044 (3)	0.049 (3)	−0.005 (2)	0.004 (2)	−0.001 (2)
C7	0.029 (2)	0.042 (3)	0.051 (3)	−0.002 (2)	0.006 (2)	0.003 (2)
C8	0.033 (2)	0.036 (2)	0.043 (2)	−0.0019 (19)	0.0078 (19)	−0.001 (2)
C9	0.034 (2)	0.031 (2)	0.044 (2)	−0.0018 (19)	0.0051 (19)	−0.0007 (19)
C10	0.035 (2)	0.034 (2)	0.043 (2)	−0.0026 (19)	0.0037 (18)	−0.0027 (19)
C11	0.036 (2)	0.048 (3)	0.042 (2)	−0.004 (2)	0.005 (2)	0.000 (2)
C12	0.034 (2)	0.038 (2)	0.047 (2)	−0.003 (2)	0.001 (2)	0.004 (2)
C13	0.039 (3)	0.036 (2)	0.035 (2)	0.000 (2)	0.0048 (18)	0.0005 (19)
C14	0.032 (2)	0.037 (2)	0.043 (2)	0.0008 (19)	0.0073 (18)	−0.002 (2)
C15	0.043 (3)	0.061 (3)	0.046 (3)	−0.004 (3)	0.012 (2)	0.000 (2)
C16	0.056 (3)	0.059 (3)	0.041 (3)	−0.005 (3)	0.008 (2)	−0.004 (2)
C17	0.043 (3)	0.040 (3)	0.041 (2)	0.000 (2)	−0.002 (2)	−0.0018 (19)
C18	0.059 (4)	0.092 (5)	0.093 (5)	0.002 (4)	0.004 (4)	0.046 (4)
C19	0.047 (3)	0.060 (3)	0.057 (3)	−0.009 (3)	0.000 (3)	0.003 (3)
C20	0.066 (4)	0.083 (4)	0.057 (3)	−0.017 (4)	−0.011 (3)	0.006 (3)
C21	0.053 (3)	0.047 (3)	0.059 (3)	0.004 (3)	0.006 (2)	−0.014 (2)
C22	0.052 (3)	0.038 (3)	0.054 (3)	0.003 (2)	0.008 (2)	0.003 (2)
O1	0.077 (3)	0.111 (4)	0.057 (2)	−0.019 (3)	0.008 (2)	−0.031 (2)
O2	0.064 (3)	0.112 (4)	0.058 (2)	−0.044 (3)	−0.007 (2)	0.005 (2)
O3	0.0452 (19)	0.063 (2)	0.0339 (15)	−0.0105 (18)	−0.0021 (13)	0.0028 (16)
O4	0.054 (2)	0.110 (4)	0.062 (2)	0.008 (3)	0.004 (2)	−0.009 (2)
O5	0.046 (2)	0.060 (2)	0.0387 (17)	−0.0038 (18)	−0.0006 (15)	−0.0044 (16)
F1	0.166 (6)	0.213 (7)	0.165 (5)	−0.031 (5)	−0.097 (5)	0.113 (5)
F2	0.093 (3)	0.092 (3)	0.200 (5)	0.032 (3)	0.061 (4)	0.049 (4)
F3	0.076 (3)	0.082 (3)	0.137 (4)	0.000 (2)	0.027 (3)	0.048 (3)

### Geometric parameters (Å, º)

**Table:**

S1—O1	1.418 (4)	C11—H11A	0.9700
S1—O2	1.422 (4)	C11—H11B	0.9700
S1—O3	1.547 (3)	C12—C13	1.528 (6)
S1—C18	1.834 (7)	C12—H12A	0.9700
C1—C2	1.542 (6)	C12—H12B	0.9700
C1—C10	1.546 (6)	C13—C17	1.502 (6)
C1—H1A	0.9700	C13—C14	1.539 (6)
C1—H1B	0.9700	C13—C22	1.549 (7)
C2—C3	1.517 (7)	C14—C15	1.541 (6)
C2—H2A	0.9700	C14—H14	0.9800
C2—H2B	0.9700	C15—C16	1.507 (7)
C3—O5	1.465 (5)	C15—H15A	0.9700
C3—C4	1.507 (7)	C15—H15B	0.9700
C3—H3	0.9800	C16—C17	1.323 (7)
C4—C5	1.518 (6)	C16—H16	0.9300
C4—H4A	0.9700	C17—O3	1.431 (6)
C4—H4B	0.9700	C18—F1	1.278 (8)
C5—C6	1.321 (6)	C18—F3	1.298 (8)
C5—C10	1.535 (6)	C18—F2	1.319 (9)
C6—C7	1.487 (6)	C19—O4	1.206 (6)
C6—H6	0.9300	C19—O5	1.350 (6)
C7—C8	1.538 (6)	C19—C20	1.487 (7)
C7—H7A	0.9700	C20—H20A	0.9600
C7—H7B	0.9700	C20—H20B	0.9600
C8—C14	1.519 (6)	C20—H20C	0.9600
C8—C9	1.543 (6)	C21—H21A	0.9600
C8—H8	0.9800	C21—H21B	0.9600
C9—C11	1.552 (6)	C21—H21C	0.9600
C9—C10	1.554 (6)	C22—H22A	0.9600
C9—H9	0.9800	C22—H22B	0.9600
C10—C21	1.552 (7)	C22—H22C	0.9600
C11—C12	1.535 (6)		
			
O1—S1—O2	122.4 (3)	C12—C11—H11B	108.5
O1—S1—O3	112.1 (2)	C9—C11—H11B	108.5
O2—S1—O3	105.7 (2)	H11A—C11—H11B	107.5
O1—S1—C18	106.9 (3)	C13—C12—C11	109.8 (4)
O2—S1—C18	106.0 (3)	C13—C12—H12A	109.7
O3—S1—C18	101.7 (3)	C11—C12—H12A	109.7
C2—C1—C10	114.9 (4)	C13—C12—H12B	109.7
C2—C1—H1A	108.5	C11—C12—H12B	109.7
C10—C1—H1A	108.5	H12A—C12—H12B	108.2
C2—C1—H1B	108.5	C17—C13—C12	119.3 (4)
C10—C1—H1B	108.5	C17—C13—C14	97.8 (4)
H1A—C1—H1B	107.5	C12—C13—C14	106.7 (4)
C3—C2—C1	108.5 (4)	C17—C13—C22	107.3 (4)
C3—C2—H2A	110.0	C12—C13—C22	111.2 (4)
C1—C2—H2A	110.0	C14—C13—C22	114.2 (4)
C3—C2—H2B	110.0	C8—C14—C13	113.3 (4)
C1—C2—H2B	110.0	C8—C14—C15	122.5 (4)
H2A—C2—H2B	108.4	C13—C14—C15	105.2 (4)
O5—C3—C4	110.7 (4)	C8—C14—H14	104.7
O5—C3—C2	107.5 (4)	C13—C14—H14	104.7
C4—C3—C2	111.0 (4)	C15—C14—H14	104.7
O5—C3—H3	109.2	C16—C15—C14	100.5 (4)
C4—C3—H3	109.2	C16—C15—H15A	111.7
C2—C3—H3	109.2	C14—C15—H15A	111.7
C3—C4—C5	110.3 (4)	C16—C15—H15B	111.7
C3—C4—H4A	109.6	C14—C15—H15B	111.7
C5—C4—H4A	109.6	H15A—C15—H15B	109.4
C3—C4—H4B	109.6	C17—C16—C15	109.4 (4)
C5—C4—H4B	109.6	C17—C16—H16	125.3
H4A—C4—H4B	108.1	C15—C16—H16	125.3
C6—C5—C4	120.7 (4)	C16—C17—O3	129.2 (4)
C6—C5—C10	123.4 (4)	C16—C17—C13	114.5 (4)
C4—C5—C10	115.8 (4)	O3—C17—C13	115.9 (4)
C5—C6—C7	126.0 (4)	F1—C18—F3	109.3 (6)
C5—C6—H6	117.0	F1—C18—F2	108.9 (7)
C7—C6—H6	117.0	F3—C18—F2	107.0 (7)
C6—C7—C8	111.3 (4)	F1—C18—S1	108.9 (7)
C6—C7—H7A	109.4	F3—C18—S1	112.4 (5)
C8—C7—H7A	109.4	F2—C18—S1	110.1 (5)
C6—C7—H7B	109.4	O4—C19—O5	122.7 (5)
C8—C7—H7B	109.4	O4—C19—C20	125.5 (5)
H7A—C7—H7B	108.0	O5—C19—C20	111.7 (5)
C14—C8—C7	110.3 (4)	C19—C20—H20A	109.5
C14—C8—C9	107.6 (4)	C19—C20—H20B	109.5
C7—C8—C9	110.3 (4)	H20A—C20—H20B	109.5
C14—C8—H8	109.6	C19—C20—H20C	109.5
C7—C8—H8	109.6	H20A—C20—H20C	109.5
C9—C8—H8	109.6	H20B—C20—H20C	109.5
C8—C9—C11	112.4 (4)	C10—C21—H21A	109.5
C8—C9—C10	112.4 (3)	C10—C21—H21B	109.5
C11—C9—C10	113.1 (4)	H21A—C21—H21B	109.5
C8—C9—H9	106.1	C10—C21—H21C	109.5
C11—C9—H9	106.1	H21A—C21—H21C	109.5
C10—C9—H9	106.1	H21B—C21—H21C	109.5
C5—C10—C1	107.9 (4)	C13—C22—H22A	109.5
C5—C10—C21	109.0 (4)	C13—C22—H22B	109.5
C1—C10—C21	110.0 (4)	H22A—C22—H22B	109.5
C5—C10—C9	109.7 (4)	C13—C22—H22C	109.5
C1—C10—C9	108.8 (4)	H22A—C22—H22C	109.5
C21—C10—C9	111.4 (4)	H22B—C22—H22C	109.5
C12—C11—C9	115.2 (4)	C17—O3—S1	124.1 (3)
C12—C11—H11A	108.5	C19—O5—C3	116.0 (4)
C9—C11—H11A	108.5		
			
C10—C1—C2—C3	−56.8 (6)	C9—C8—C14—C13	61.6 (5)
C1—C2—C3—O5	−179.3 (4)	C7—C8—C14—C15	−50.3 (6)
C1—C2—C3—C4	59.5 (6)	C9—C8—C14—C15	−170.6 (4)
O5—C3—C4—C5	−178.0 (4)	C17—C13—C14—C8	169.8 (4)
C2—C3—C4—C5	−58.7 (5)	C12—C13—C14—C8	−66.4 (5)
C3—C4—C5—C6	−123.9 (5)	C22—C13—C14—C8	56.8 (5)
C3—C4—C5—C10	54.4 (6)	C17—C13—C14—C15	33.5 (5)
C4—C5—C6—C7	−178.1 (5)	C12—C13—C14—C15	157.3 (4)
C10—C5—C6—C7	3.8 (8)	C22—C13—C14—C15	−79.6 (5)
C5—C6—C7—C8	13.3 (7)	C8—C14—C15—C16	−164.6 (4)
C6—C7—C8—C14	−162.7 (4)	C13—C14—C15—C16	−33.4 (5)
C6—C7—C8—C9	−44.0 (5)	C14—C15—C16—C17	19.7 (6)
C14—C8—C9—C11	−49.8 (5)	C15—C16—C17—O3	175.0 (5)
C7—C8—C9—C11	−170.1 (4)	C15—C16—C17—C13	2.1 (6)
C14—C8—C9—C10	−178.7 (4)	C12—C13—C17—C16	−136.8 (5)
C7—C8—C9—C10	61.0 (5)	C14—C13—C17—C16	−22.7 (5)
C6—C5—C10—C1	129.6 (5)	C22—C13—C17—C16	95.7 (5)
C4—C5—C10—C1	−48.6 (5)	C12—C13—C17—O3	49.2 (6)
C6—C5—C10—C21	−111.0 (5)	C14—C13—C17—O3	163.4 (4)
C4—C5—C10—C21	70.8 (5)	C22—C13—C17—O3	−78.2 (5)
C6—C5—C10—C9	11.2 (6)	O1—S1—C18—F1	72.5 (6)
C4—C5—C10—C9	−167.0 (4)	O2—S1—C18—F1	−59.5 (6)
C2—C1—C10—C5	50.1 (5)	O3—S1—C18—F1	−169.8 (5)
C2—C1—C10—C21	−68.7 (5)	O1—S1—C18—F3	−48.9 (6)
C2—C1—C10—C9	169.0 (4)	O2—S1—C18—F3	179.2 (5)
C8—C9—C10—C5	−42.9 (5)	O3—S1—C18—F3	68.9 (6)
C11—C9—C10—C5	−171.4 (4)	O1—S1—C18—F2	−168.1 (5)
C8—C9—C10—C1	−160.7 (4)	O2—S1—C18—F2	59.9 (6)
C11—C9—C10—C1	70.7 (5)	O3—S1—C18—F2	−50.4 (6)
C8—C9—C10—C21	77.8 (5)	C16—C17—O3—S1	−12.4 (7)
C11—C9—C10—C21	−50.7 (5)	C13—C17—O3—S1	160.5 (3)
C8—C9—C11—C12	48.0 (6)	O1—S1—O3—C17	38.7 (5)
C10—C9—C11—C12	176.5 (4)	O2—S1—O3—C17	174.3 (4)
C9—C11—C12—C13	−51.9 (6)	C18—S1—O3—C17	−75.2 (4)
C11—C12—C13—C17	167.1 (4)	O4—C19—O5—C3	−1.4 (8)
C11—C12—C13—C14	57.8 (5)	C20—C19—O5—C3	178.3 (4)
C11—C12—C13—C22	−67.2 (5)	C4—C3—O5—C19	−79.5 (5)
C7—C8—C14—C13	−178.1 (4)	C2—C3—O5—C19	159.1 (4)

### Hydrogen-bond geometry (Å, º)

**Table:**

*D*—H···*A*	*D*—H	H···*A*	*D*···*A*	*D*—H···*A*
C1—H1*A*···O4^i^	0.97	2.56	3.485 (6)	160
C21—H21*B*···O2^ii^	0.97	2.65	3.377 (7)	133

Symmetry codes: (i) *x*−1, *y*, *z*; (ii) −*x*, *y*+1/2, −*z*+1/2.
