# Supplementary figures and images for: Crystal structure of 3β-acet­oxy­androsta-5,16-dien-17-yl tri­fluoro­methane­sulfon­ate
Source: Acta Crystallogr E Crystallogr Commun. 2015 May 16;71(Pt 6):o404–5. doi: 10.1107/S2056989015009123 (PMC4459318; doi:10.1107/S2056989015009123)

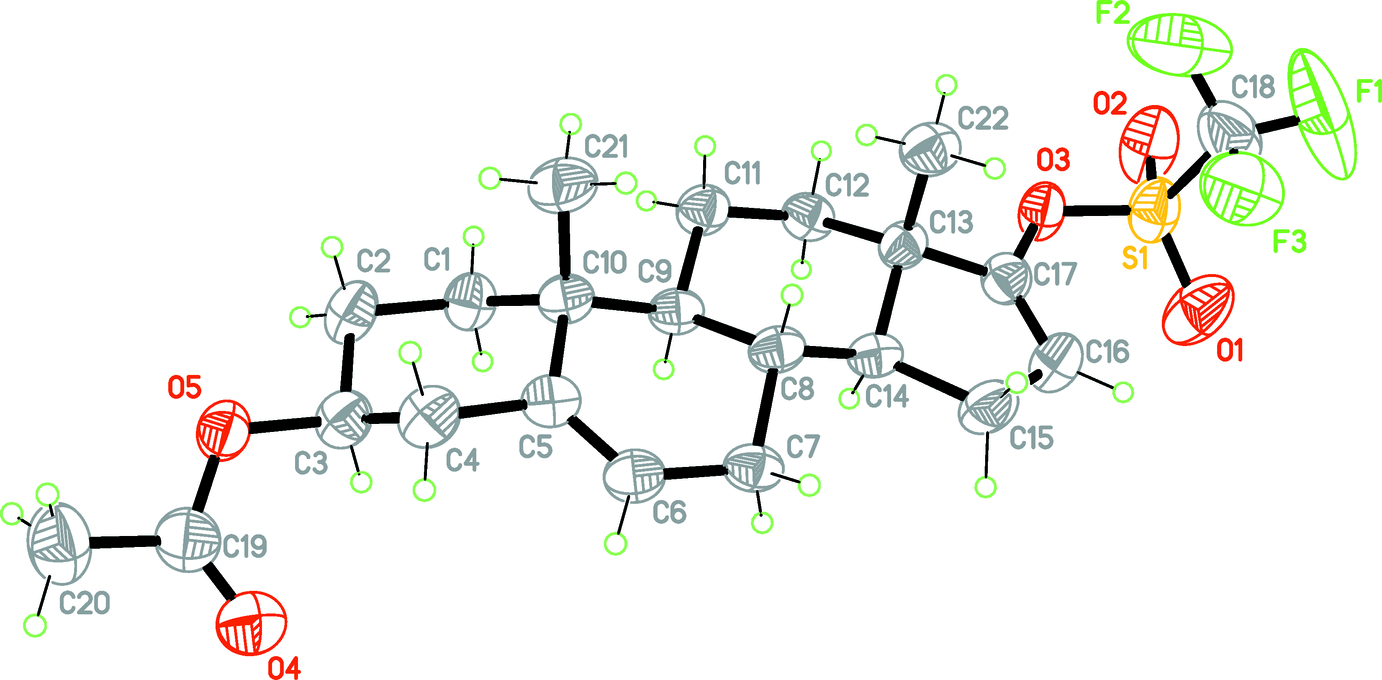

Supplement: Supplementary file 4 [file e-71-0o404-fig1.tif]
